# The deadly impact of COVID-19 among children from Latin America: The case of Ecuador

**DOI:** 10.3389/fped.2023.1060311

**Published:** 2023-04-21

**Authors:** Esteban Ortiz-Prado, Juan S. Izquierdo-Condoy, Raul Fernandez-Naranjo, Jorge Vasconez, María Gabriela Dávila Rosero, Doménica Revelo-Bastidas, Diva Herrería-Quiñonez, Mario Rubio-Neira

**Affiliations:** ^1^ One Health Research Group, Faculty of Health Science, Universidad de Las Américas, Calle de los Colimes y Avenida De los Granados, Quito, Ecuador; ^2^ Facultad de Ciencias de la Salud, Universidad Latina de Costa Rica, San José, Costa Rica; ^3^ Department of Cardiology, Baca Ortiz Pediatric Hospital, Quito, Ecuador

**Keywords:** COVID-19, SARS-CoV-2, children, pediatric population, adolecent, epidemiology, latinamerica, Ecuador (country)

## Abstract

**Background:**

The SARS-CoV-2 pandemic remains a critical global health concern, with older adults being the most vulnerable group. Nonetheless, it is crucial to recognize that COVID-19 has caused numerous deaths in children worldwide. Emerging evidence indicates that infants and breastfeeding children, particularly those aged below one year, face a greater risk of hospitalization and mortality than older children with COVID-19.

**Objective:**

This study aimed to describe the epidemiology of COVID-19 among children during the early phase of the pandemic in Ecuador.

**Methods:**

We conducted a country-wide population-based analysis of the epidemiology of COVID-19, using incidence and mortality data reported from Ecuador between February 15, 2020 and May 14 2021. Measurements of frequency, central tendency, dispersion, and absolute differences were calculated for all categorical and continuous variables.

**Results:**

At least 34,001 cases (23,587 confirmed cases, 5,315 probable and 5,099 suspected) and 258 COVID-19 related deaths have been reported among children in Ecuador during the first 16 months of the pandemic. The overall incidence rate was 612 cases per 100,000 children, the mortality rate was 3 per 100,000, while the case fatality rate was 0.76%. The highest risk group for infection was children and adolescents between 15 and 19 years of age; however, the highest mortality rate occurred in children under one year of age. The largest provinces, such as Pichincha, Guavas and Manabí, were the ones that reported the highest number of cases, 27%, 12.1% and 10.8%, respectively.

**Conclusions:**

This study is the first to report on COVID-19 epidemics among children in Ecuador. Our findings reveal that younger children have a lower risk of SARS-CoV-2 infection, but a higher risk of mortality compared to older children and adolescents. Additionally, we observed significant disparities in infection rates and outcomes among children living in rural areas, those with comorbidities, and those from indigenous ethnic groups.

## 1. Introduction

Since December 2019, at least 658 million cases and arguable more than 6.6 million deaths have been attributed to the SARS-CoV-2 virus (1–5). In the early phase of the pandemic, we learned that the most affected populations were the elderly, those with comorbidities, and those with adverse social determinants (6). However, with the deployment of vaccines, the unvaccinated and those with incomplete vaccination schemes became the most affected population in terms of severity and mortality (7). While there are increasing reports globally on the effects of COVID-19 among children and adolescents, there is still limited knowledge on the impact of the disease on children in developing countries (8).

Since the first pediatric case of COVID-19 was reported on January 20, 2020 in China, there have been numerous reports worldwide attempting to address the issue of COVID-19 among this population (9–11). Evidence suggests that, in comparison to older populations, children are less likely to die from COVID-19, exhibit significantly lower case fatality rates, experience milder symptoms, and have a quicker recovery time than adults (12–15). Nonetheless, children comprise between 1% and 2% of the overall pool of positive cases and 0.4% of the total number of COVID-19 related deaths worldwide (16). Although current literature confirms that among adults, the proportion of deaths is equal between men and women, deaths are more likely to occur in men. In children, however, it seems like younger female children might have a slightly higher risk of dying and a consistent positive correlation between age and the likelihood of getting COVID-19 (17–19).

**Figure 1:**
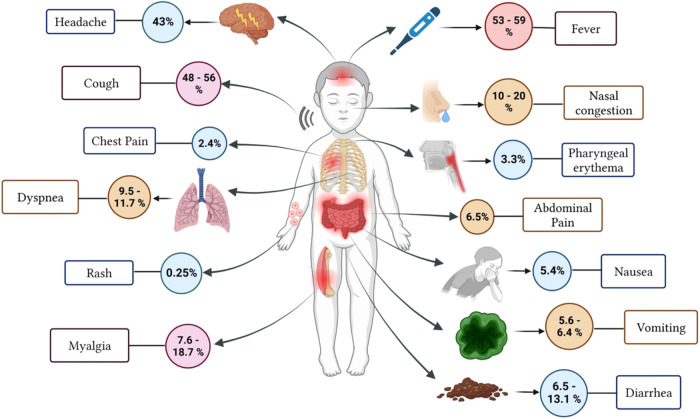
Estimated frequency of Symptoms of COVID-19 infection in pediatric patients (Adapted from Borelli et al., 2021).

According to Patel's et.al systematic review, there is an increased risk of illness among older children, although mortality rates decrease as the child enters adolescence (19). Viner et al., 2021, reported that children without symptoms made up anywhere from 14.6% to 42% of the studied population (20). The most prevalent symptoms were also fever, varying from 46% to 64.2% and cough, from 32% to 55.9% (20). The frequency of all other symptoms and indicators, such as rhinorrhea, headache, fatigue/myalgia, and gastrointestinal problems including diarrhea and vomiting, represented from 10% to 20% of the pediatric population (20) (Figure 1).

In terms of mortality rates, children are less likely to develop severe cases, nevertheless, reports worldwide vary from 0.1% up to 1% (21, 22). Data suggest that newborns and children, especially those under one year of age are more likely to develop severe disease (21, 22). Like adults, children with comorbidities have a higher risk for developing symptomatic COVID-19 infection and have poorer prognosis than healthy children. Shekerdemian et al. (2020) found that 40 out of 48 children who required life support within the pediatric intensive care unit (PICU) had underlying conditions (genetic abnormalities, immunosuppression, cancer, malignancy, other congenital heart disease, obesity and chronic kidney diseases) (23). The epidemiological, sociodemographic, and clinical features of children with COVID-19 are known within the develop world; however, very few investigations involving children from low and middle-income countries, particularly from Latin America have been published.

The objective of this study was to describe the epidemiology of COVID-19 among Ecuadorian children.

## 2. Methodology

### 2.1. Study design

We conducted a country-wide population-based analysis of the epidemiology of COVID-19 among children from 0 to 19 years of age.

### 2.2. Setting

The study was conducted in Ecuador, a country located in South America, bordering with Colombia to the North, Peru to the South/East and the Pacific Ocean to the West. The 2020 population projections estimated a total of 17.5 million people for 2020, having a pediatric and adolescent population of 5.9 million (24).

### 2.3. Data sources and description

A country-wide comparison of the data from the 24 provinces and 221 cantons was performed using the complete dataset from the Ministry of Public Health's national databases on overall mortality and hospital discharge data from February 2020 to May 2021. The 2020 population census data from the National Institute of Census and Statistics (INEC) were used to compute age and sex adjusted rates.

All cases of COVID-19 were classified according to the International Classification of Diseases 10th Revision (ICD-10). ICD-10 codes reflecting intentional self-harm (U07) were included in the analysis.

Cases were identified according to guidelines from the Ministry of Public Health, which define three categories of cases.

A case was identified as confirmed by a positive RT-PCR test, regardless of symptoms, or a positive rapid antigen detection test and meeting the criteria for a probable or suspected case. This included asymptomatic or symptomatic individuals who have had contact with a confirmed or probable case and test positive.

A case was defined as probable when a person met the clinical criteria for a suspected case and had been a contact of a confirmed or probable case, had imaging findings suggestive of COVID-19 disease, had recent onset of anosmia or ageusia or respiratory symptoms without another explanation, or has unexplained death with respiratory distress and had contact with a confirmed or probable case or were linked to an identified COVID-19 cluster.

A suspected case was identified by meeting the clinical criteria, including an acute onset of fever and cough, as well as three or more of several other symptoms such as headache, sore throat, or gastrointestinal symptoms. The epidemiological criteria included residing or working in an area with a high risk of virus transmission, having high-risk contact with a confirmed or probable case of COVID-19, traveling to a country with community transmission, or working in a healthcare facility.

The presence of comorbidities was assessed based on the diseases and conditions reported by patients, including but not limited to: arterial hypertension, chronic kidney disease, asthma, diabetes, chronic hepatic disease, psoriasis, cancer, chronic vascular disease, COPD, rheumatoid arthritis, tuberculosis, hepatic cirrhosis, pulmonary fibrosis, obesity, and hypothyroidism.

### 2.4. Data analysis

The overall incidence and mortality rate, as well as age and sex specific case fatality rate (CFR) percentage were computed according to the entire children and adolescent population at risk (by canton and province). Measurements of frequency (counts, absolute and relative percentages), central tendency (median), dispersion (interquartile range) and absolute differences were calculated for all categorical and continuous variables using a methodology previously reported (25). In our study, we computed the CFR by using the number of cases officially reported as COVID-19 related deaths.

In our analysis, we considered deaths as a binary variable, meaning that individuals were categorized as either alive or dead. We used this variable as an outcome to calculate the CFR percentage, which is determined by dividing the number of deaths reported as COVID-19 related by the number of confirmed COVID-19 cases.

We used two-pronged approach to analyze the multivariate relationship between infection and death rates. First, we conducted an ANOVA test on linear models that consider both cases and deaths. This helped us to identify the variables that were associated with these features. Second, we conducted a principal component analysis of all variables to determine which ones were related to the main features of cases and deaths. Through these methods, we aimed to gain a deeper understanding of the factors that contributed to infection and death rates.

We analyzed and described every variable using the IBM SPSS statistics version 24.0. We designed figures and other graphics using the prism 9 data visualization software and Piktochart infographic online app. References and citations were managed by Zotero Open-Source Software version 4.0.11.

### 2.5. Study size and sample size calculation

We have included every confirmed, probable, and suspected COVID-19 case reported to the Public Health Surveillance System (ViEpi) in Ecuador. According to the MoH of Ecuador, during the first 454 days of the duration of the pandemic (February 15th, 2020 to May 14th, 2021), a total of 473,937 cases and 23, 803 COVID-19 related deaths were reported in Ecuador. From these, 34,001 cases and 258 COVID-19 related deaths were registered among children and adolescents.

### 2.6. Ethics statement

This study involved a secondary data analysis of anonymized and unidentifiable information, which received ethical approval (#EOP-200301–001) from the Universidad de las Americas Ethics Committee CEISH on March 10th, 2020. To ensure data privacy and confidentiality, the Minister of Public Health robustly anonymized the data, and no personal identifiable data was shared. We obtained authorization to access the countrywide level data from the former Minister of Health through N. MSP-MSP-2021-1523-O. Our research was conducted in compliance with local and international ethical standards and all ethical principles for medical research involving human subjects, including the Helsinki Declaration. We took every measure to avoid potential ethical issues and prevent any selection or information bias that could compromise the integrity of our findings.

### 2.7. Bias

To minimize selection and information bias, we obtained the secondary dataset directly from the MoH shared file. EOP, RFN, and JSI analyzed the data independently, and in cases where the results were not consistent, we reviewed the entire dataset and considered automated measures and appropriate data codification to prevent potential human errors. Through these measures, we aimed to ensure the accuracy and reliability of our findings.

## 3. Results

In Ecuador, during 454 days of the total analysis period, from February 15, 2020 to May 14, 2021, at least 34,001 cases and 258 COVID-19 related deaths were registered among children and adolescents from 0 to 19 years of age within the national surveillance system.

### 3.1. Sex differences

Within all the age categories, at least 17,336 (51%) cases were reported among the female pediatric population and 16,665 (49%) among males (Table 1)

**Table 1:** Female and male COVID-19 cases reported among children and adolescents in Ecuador from 2020 to 2021.

Age category	Sex	Confirmed cases	Probable cases	Suspected cases		COVID-19 cases	Discarded cases			Deaths
		*N*	*n*	*N*	*n*	(%)	*n*	(%)	*n*	(%)
0–4 years	Female	1,052	467	524	2,043	47%	5,689	47%	70	48%
	Male	1,157	547	581	2,285	53%	6,494	53%	77	52%
5–9 years	Female	1,464	390	360	2,214	48%	4,909	48%	17	52%
	Male	1,531	446	398	2,375	52%	5,226	52%	16	48%
10–14 years	Female	3,082	637	539	4,258	50%	7,504	50%	15	44%
	Male	3,002	621	576	4,199	50%	7,387	50%	19	56%
15–19 years	Female	6,480	1,259	1,082	8,821	53%	16,935	54%	25	57%
	Male	5,819	948	1,039	7,806	47%	14,274	46%	19	43%
Subtotal	Female	12,078	2,753	2,505	17,336	51%	35,037	51%	127	49%
	Male	11,509	2,562	2,594	16,665	49%	33,381	49%	131	51%
**Total**	** **	**23,587**	**5,315**	**5,099**	**34,001**	**100%**	**68,418**	**100%**	**258**	**100%**

### 3.2. Age differences

Among the analyzed cases, the median age for males was 14 years (IQR 9–17 years) and 15 years (IQR 10–17 years) for females. While regarding deaths recorded, the median age in the male group was one year (IQR 0–11.5 years) and two years (IQR 0–12.5 years) for females.

In Ecuador, at least 14.9% (*n* = 5,054) of the COVID-19 new cases were reported among pre-scholar group (0 to 5 years), 2,392 (47.3%) in female children and 2,662 (52.7%) among males. Through the adolescents' group (12 to 18 years), the subgroup with the highest risk of infection is over 16 years of age, who combined account for 41.6% (*n* = 14,154) of the entire cohort, 7,468 female (21.9%) and 6,686 (19.6%) male adolescents (Table 2).

**Table 2:** Age related sex differences between children and adolescents from 0 to 19 years of age.

Age (year)	Female				Male				
	*n*	(%)	Deaths	CFR	*n*	(%)	Deaths	CFR	CFR Diff.
0	645	4%	57	8.8%	785	5%	57	7.3%	1.6%
1	404	2%	6	1.5%	473	3%	12	2.5%	−1.1%
2	328	2%	1	0.3%	365	2%	4	1.1%	−0.8%
3	337	2%	3	0.9%	339	2%	1	0.3%	0.6%
4	329	2%	3	0.9%	323	2%	3	0.9%	0.0%
5	349	2%	5	1.4%	377	2%	5	1.3%	0.1%
6	388	2%	7	1.8%	433	3%	1	0.2%	1.6%
7	412	2%	0	0.0%	472	3%	2	0.4%	−0.4%
8	509	3%	3	0.6%	514	3%	4	0.8%	−0.2%
9	556	3%	2	0.4%	579	3%	4	0.7%	−0.3%
10	598	3%	1	0.2%	664	4%	3	0.5%	−0.3%
11	730	4%	2	0.3%	706	4%	2	0.3%	0.0%
12	843	5%	5	0.6%	840	5%	2	0.2%	0.4%
13	959	6%	4	0.4%	919	6%	6	0.7%	−0.2%
14	1,128	7%	3	0.3%	1,070	6%	6	0.6%	−0.3%
15	1,353	8%	4	0.3%	1,120	7%	5	0.4%	−0.2%
16	1,607	9%	4	0.2%	1,337	8%	4	0.3%	−0.1%
17	1,820	10%	6	0.3%	1,538	9%	3	0.2%	0.1%
18	1,865	11%	4	0.2%	1,748	10%	2	0.1%	0.1%
19	2,176	13%	7	0.3%	2,063	12%	5	0.2%	0.1%
**Total**	**17,336**	**100%**	**127**	**0.7%**	**16,665**	**100%**	**131**	**0.8%**	**−0.1%**

CFR, case fatality rate.

### 3.3. Mortality and CFR (%) among children and adolescents

**Figure 2:**
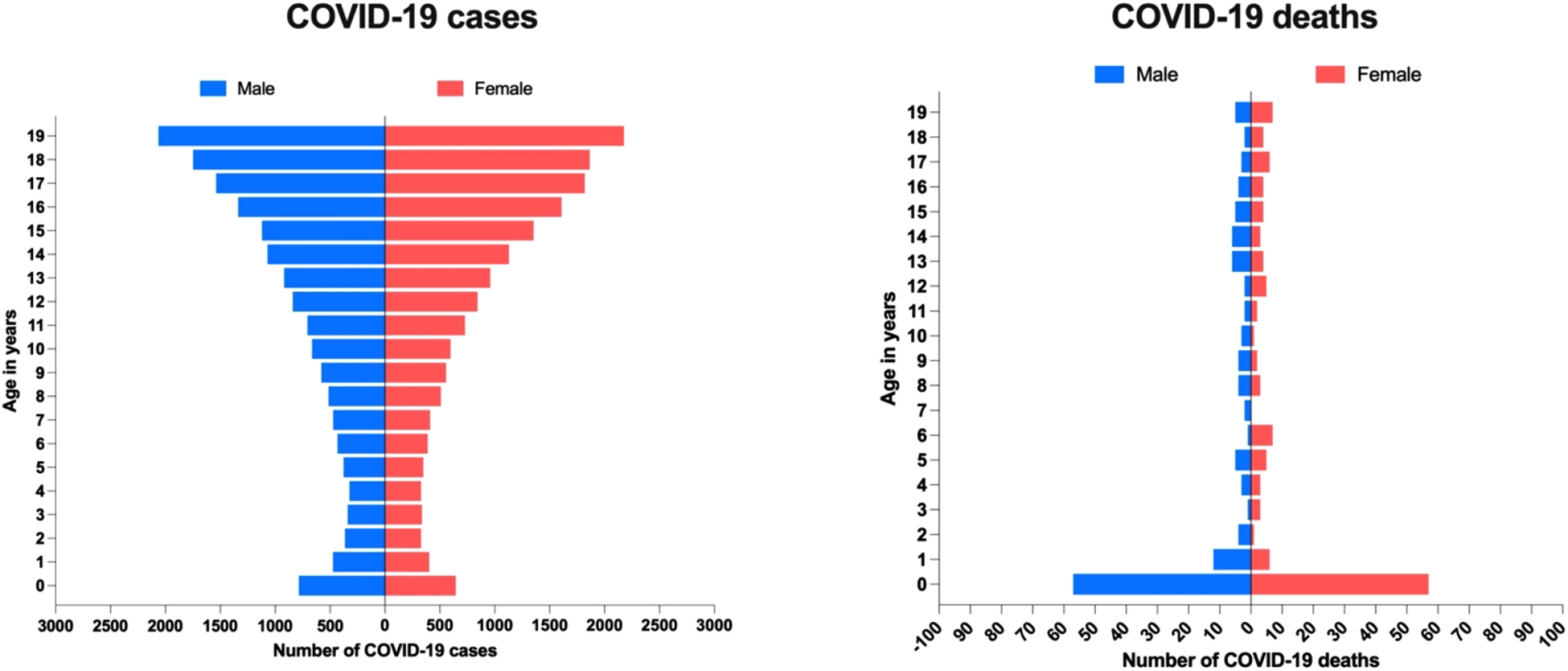
COVID-19 related cases and deaths by age among pediatric and adolescent population in Ecuador.

During the first 454 days of the pandemic in Ecuador, 127 female (49%) and 131 male (51%) children and adolescents died due to COVID-19. In the same period, we found that most deaths were reported among children from 0 to 1 year of age, representing 44% (*n* = 114) of the total number of deaths reported (Figure 2).

### 3.4. Risk analysis among children and adolescents with COVID-19 in Ecuador

After using the population with the lowest risk of dying from COVID-19 (15 to 19 years old) as a reference, we found that infants (less than one-year-old) have the highest odds of dying from COVID-19 among the entire pediatric population (Table 3).

**Table 3:** Analysis of risk and probability of infection and death among children and adolescents with COVID-19 in Ecuador.

		Sex	Cases *n*	Deaths *n*	CFR %	OR	CI 95%	*P* Value
Age (years)	0–4	Female	2,043	70	3.4%	12.5	7.88–19.75	**0**.**0001**
		Male	2,285	77	3.4%	14.3	8.63–23.66	**0**.**0001**
	5–9	Female	2,214	17	0.8%	2.7	1.46–5.05	**0**.**0015**
		Male	2,375	16	0.7%	2.8	1.42–5.41	**0**.**0026**
	10–14	Female	4,258	15	0.4%	1.2	0.65–2.36	0.500
		Male	4,199	19	0.5%	1.9	0.98–3.52	0.055
	15–19	Female	8,821	25	0.3%	ref	ref	ref
		Male	7,806	19	0.2%	ref	ref	ref
Comorbidities	No	Female	16,883	83	0.5%	ref	ref	ref
		Male	16,209	97	0.6%	ref	ref	ref
	Yes	Female	453	44	9.7%	21.8	14.92–31.79	**<0.0001**
		Male	456	34	7.5%	12.6	8.35–18.99	**<0.0001**
Ethnicity	Afro	Female	83	2	2.4%	3.8	0.88–14.99	0.0661
		Male	68	0	NA	1.1	0.06–17.07	0.9725
	White	Female	36	0	NA	2.1	0.12–34.18	0.6071
		Male	39	0	NA	1.8	0.11–29.83	0.6743
	Indigenous	Female	542	4	0.7%	1.1	0.41–3.09	0.8026
		Male	447	10	2.2%	3.3	1.71–6.38	**0**.**0004**
	Mestizo	Female	15,232	99	0.6%	ref	ref	ref
		Male	14,560	100	0.7%	ref	ref	ref
	Montubio	Female	127	1	0.8%	1.2	0.16–8.76	0.8481
		Male	116	0	NA	0.6	0.03–10.00	0.7344
	Mulato	Female	13	0	NA	5.6	0.33–95.41	0.2311
		Male	14	0	NA	4.9	0.29–83.74	0.2666
	Other	Female	1,303	21	1.6%	2.5	1.55–4.02	**0**.**0001**
		Male	1,421	21	1.5%	2.2	1.35–3.48	**0**.**0014**
**Total**	** **		34,001	258	0.8%			

CFR, case fatality rate; OR, Odds ratio; OR, Odds ratio; CI 95%, Confidence intervals.

Male and female children with comorbidities showed a higher risk of dying from COVID-19 (OR: 12.6; 95% CI: 8.35–18.99, and OR: 21.8; 95% CI: 14.92–31.79, respectively).

We found that the CFR is 305% higher among infants younger than 12 months of age than those older than one year, 1,057% higher than those from two to three years, 1,250% from those three to four years, and 3,140% higher than 19 years old adolescents (Table 2). In comparison with adults, CFR increases dramatically with age, especially after the age of 50, where CFR increases from 5,5% at 55 years up to almost 20% when reaching the late 80's (Supplementary File S1).

Our findings from a multivariate analysis conducted on the risk of infection and mortality among individuals. Our results indicate that ethnicity and sex did not show any significant association with the risk of infection, as measured by infection rate. However, we found that the presence of a comorbidity was positively associated with the likelihood of becoming infected among confirmed cases (Table 4).

**Table 4:** Pediatric COVID-19 cases and deaths per province.

Province	Cases (*n*)	Deaths (*n*)	Pop. At Risk (*n*)	CFR (%)	IR per 100,000	MR per 100,000	Cases (%)	Deaths (%)
Azuay	1,716	7	318,092	0.4%	539	2	5.0%	2.7%
Bolivar	732	1	91,892	0.1%	797	1	2.2%	0.4%
Cañar	352	0	113,906	0.0%	309	0	1.0%	0.0%
Carchi	917	1	68,087	0.1%	1,347	1	2.7%	0.4%
Chimborazo	411	7	203,710	1.7%	202	3	1.2%	2.7%
Cotopaxi	906	2	203,155	0.2%	446	1	2.7%	0.8%
El Oro	1,910	7	259,299	0.4%	737	3	5.6%	2.7%
Esmeraldas	787	3	288,605	0.4%	273	1	2.3%	1.2%
Galapagos	207	0	12,284	0.0%	1,685	0	0.6%	0.0%
Guayas	4,131	31	1,596,161	0.8%	259	2	12.1%	12.0%
Imbabura	1,477	6	181,429	0.4%	814	3	4.3%	2.3%
Loja	1,500	6	199,990	0.4%	750	3	4.4%	2.3%
Los rios	1,081	5	373,930	0.5%	289	1	3.2%	1.9%
Manabi	3,666	24	600,199	0.7%	611	4	10.8%	9.3%
Morona santiago	816	3	95,154	0.4%	858	3	2.4%	1.2%
Napo	353	11	61,889	3.1%	570	18	1.0%	4.3%
Orellana	291	0	76,976	0.0%	378	0	0.9%	0.0%
Pastaza	398	5	50,248	1.3%	792	10	1.2%	1.9%
Pichincha	9,189	117	1,115,305	1.3%	824	10	27.0%	45.3%
Santa elena	301	2	166,342	0.7%	181	1	0.9%	0.8%
Santo Domingo	1,076	12	182,081	1.1%	591	7	3.2%	4.7%
Sucumbios	603	4	97,474	0.7%	619	4	1.8%	1.6%
Tungurahua	1,000	3	205,905	0.3%	486	1	2.9%	1.2%
Zamora Chinchipe	181	1	55,412	0.6%	327	2	0.5%	0.4%
**Total**	**34,001**	**258**	**6,617,525**	**0.6%**	**612**	**3**	**100.0%**	**100.0%**

CFR, case fatality rate; IR, incidence rate; MR, mortality rate.

### 3.5. Geographical differences

In Ecuador, the largest provinces, such as Pichincha, Guavas, Manabí, were those where the highest number of cases were reported, i.e., 27%, 12.1% and 10.8%, respectively. However, in terms of CFR percentage, it is noteworthy that the province of Napo and Chimborazo were the ones that reported the highest mortality, 3.1% and 1.7%, respectively. In terms of infections per population at risk, the province of Galapagos (1,685/100,000), followed by Carchi (1,347/100,000) and Morona Santiago (858/100,000), reported the highest number of cases per 100,000 inhabitants. Concerning mortality per 100,000 inhabitants, Napo (18/100,000), followed by Pichincha (10/100,000) and Pastaza (10/100,000), were the provinces with the highest number of cases per 100,000 inhabitants (Table 5).

**Table 5:** Multivariate analysis.

Independent Variable		COVID-19 cases				COVID-19 Deaths			
		df	Mean Sq	*F*	Sig	df	Mean Sq	*F*	Sig
Comorbidities	No	1	773,723	47.691	.**0001**	1	192.734	2.626	.**0001**
	Yes	1	1,244	7.667	.**0001**	1	68.353	9.313	.**0001**
Ethnic Category	Mestizo	1		2.034	0.652	1		0.000	0.9989
	Other	1		42.200	0.924	1		50.243	0.9828
	Montubio	1		8.600	0.926	1		50.243	0.9436
	Indigenous	1		92.030	0.923	1		0.000	0.9885
	Afro-Ecuadorian	1		13.543	0.907	1		0.000	0.9832
Age	Age	1		4.222	**0**.**040**	1		1.000	0.9204
	Residuals	438				439			

**Figure 3:**
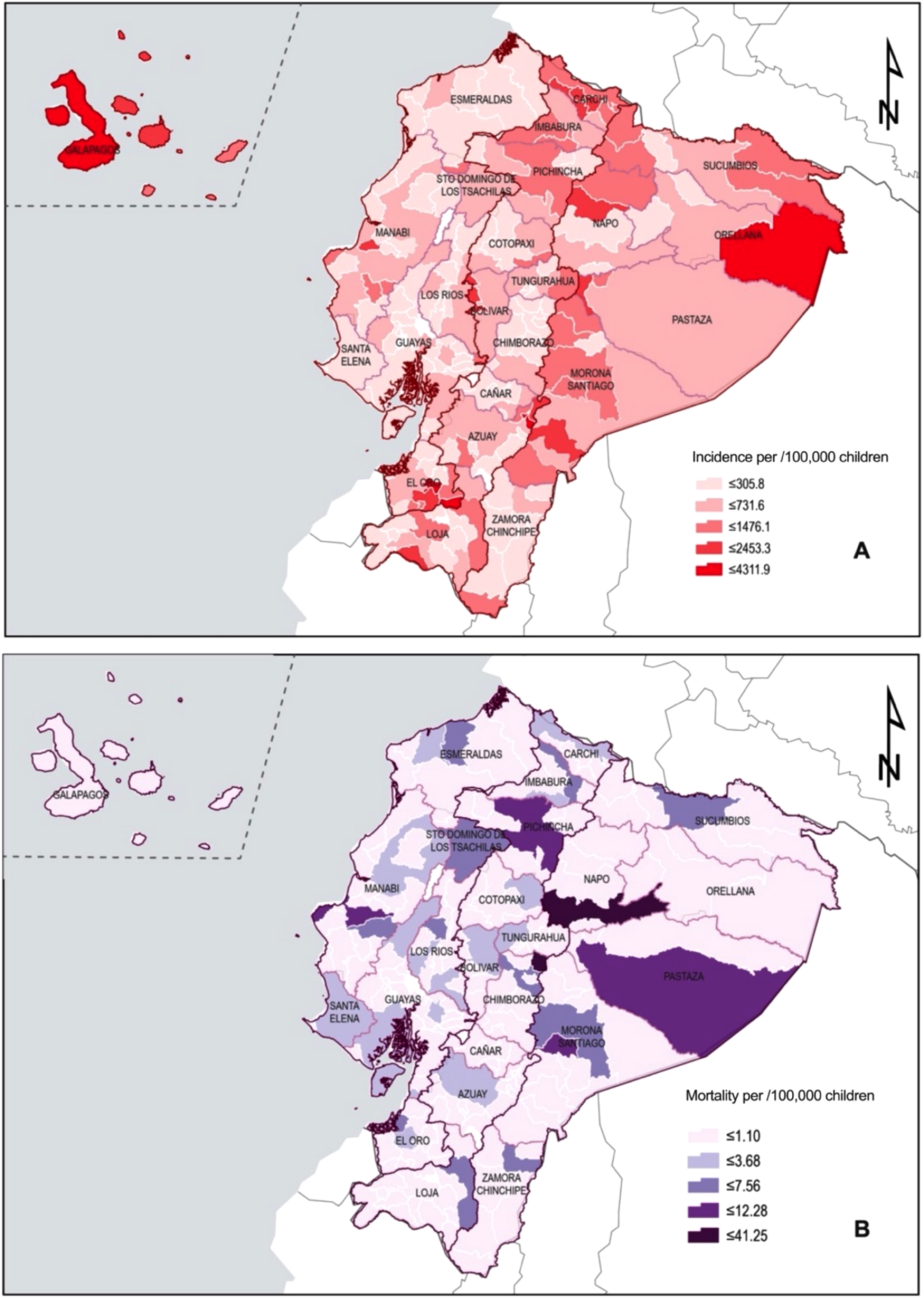
Geographic distribution of COVID-19 cases and deaths among Ecuadorian children. (**A**) Incidence rate by cantons. (**B**) Mortality rate by cantons.

The geographic analysis revealed that among pediatric patients, the highest rate of COVID-19 infection was observed in Atahualpa (4,312/100,000), Guachapala (3,995/100,000), Portobelo (3,784/100,000), Aguarico (3,712/100,000), and Isabela in the Galapagos Islands with 3,382/100,000 (as shown in Figure 3A). Meanwhile, in terms of mortality by canton, Penipe had the highest mortality rate (41/100,000), followed by Tena (27/100,000) and Pastaza (12/100,000) (as shown in Figure 3B).

## 4. Discussion

To the best of our knowledge this is the first epidemiological report of COVID-19 among Ecuadorian children and adolescent. We found a high incidence and mortality rate within this population during the early phase of the pandemic. A total of all 34,001 cases (confirmed, probable, suspected) and 258 COVID-19 related deaths were reported in the pediatric population, a significantly higher burden than the one reported elsewhere (26, 27).

Within the Latin-American context, only Brazil reported a greater CFR than Ecuador. Oliveira et. Al found that among 11,613 children infected with SARS-CoV-2, at least 886 children died due to COVID-19 (CFR 7.6%) (28), however, the overall crude mortality rate in Ecuador (1.4/100,000) was three times higher than in Brazil (0.4/100,000). In the neighboring country of Colombia, a 0.16% CFR was found (29). If we compare our results with European children, Ecuador reported a much higher CFR than Spain (CFR = 0.02%) (30), or Italy (CFR = 0.10%) (31).

During the study period in Ecuador, the predominant variant of the COVID-19 virus was the ancestral variant, with other variants such as Alpha, Gamma, Delta, Iota, Lambda, and Mu appearing in lower percentages as of January 2021 (Table 6). However, the fatality rates in the pediatric population associated with the different variables did not show significant differences after the arrival of variants other than the ancestral variant (Supplementary File S2) (32).

**Table 6:** SARS-CoV-2 variants circulating in Ecuador from 2020 to May 2021.

	Month	SARS-CoV-2 variant (%)						Pediatric COVID-19		
		Ancestral	Alpha	Gamma	Iota	Lambda	Mu	Cases (*n*)	Deaths (*n*)	Fatality rate (%)
2020	February	100.0	0.0	0.0	0.0	0.0	0.0	16	3	18.8
	March	100.0	0.0	0.0	0.0	0.0	0.0	529	25	4.7
	April	100.0	0.0	0.0	0.0	0.0	0.0	797	26	3.3
	May	100.0	0.0	0.0	0.0	0.0	0.0	1,394	29	2.1
	June	100.0	0.0	0.0	0.0	0.0	0.0	2,499	18	0.7
	July	100.0	0.0	0.0	0.0	0.0	0.0	3,632	22	0.6
	August	100.0	0.0	0.0	0.0	0.0	0.0	3,004	16	0.5
	September	100.0	0.0	0.0	0.0	0.0	0.0	2,381	19	0.8
	October	100.0	0.0	0.0	0.0	0.0	0.0	1,954	13	0.7
	November	100.0	0.0	0.0	0.0	0.0	0.0	1,698	17	1.0
	December	100.0	0.0	0.0	0.0	0.0	0.0	1,828	21	1.1
2021	January	80.5	14.6	0.0	4.9	0.0	0.0	2,703	15	0.6
	February	100.0	0.0	0.0	0.0	0.0	0.0	3,033	13	0.4
	March	63.2	16.8	8.4	9.5	2.1	0.0	3,558	17	0.5
	April	57.1	17.1	3.4	15.6	6.3	0.5	3,794	3	0.1
	May	56.4	11.8	3.5	18.1	8.4	1.9	1,181	1	0.1

It is possible that the lack of natural immunity to the virus and the absence of pre-existing treatments or vaccines contributed to the high infectiousness of the ancestral variant, which made COVID-19 so lethal at the beginning of the pandemic (33). In addition, overwhelmed healthcare systems and the vulnerability of certain populations also contributed to the severity of the pandemic.

The burden of COVID-19 among children is significantly lower than adults and the elderly. In terms of symptomatology and severity, milder cases are often compared to common endemic viral infections of childhood such as those caused by adenovirus or respiratory syncytial virus (34, 35). The reasons behind differences in severity and prognosis between children and adults could be explained by several factors (21). Zimmermann and Curtis, 2020, have summarized them in: age-related endothelial changes, higher density and increased affinity for transmembrane serine protease-2 and angiotensin converting enzyme-2 receptors; pre-existing coronavirus antibodies and T cells as well as immunosenescence and lower viral loads due to host-specific factors (36). Although most of the few available epidemiological analyses on COVID-19 in children have shown that fatal outcome of the infection occurs in very low frequencies (12, 30, 37), in this study we observed that in Ecuador, the mortality triggered by SARS-CoV-2 in children had a dramatic toll on deaths per 100,000 people, comparable only with those reported in Brazil (28).

In adults the sex-linked effects of COVID-19 are characterized by similar infection rates between males and females, and more severe cases along with mortality in males (38, 39), on the other hand, no sex-related differences in COVID-19 mortality have been demonstrated in children (29, 37, 40–42). In this context, in Ecuadorian children, the overall incidence and mortality from COVID-19 between males and females was very close; however, in 1-year-old girls, the CFR was slightly higher in boys (Table 2).

Regarding the age of the pediatric population, the highest number of COVID-19 cases was observed in populations older than 15 years. This high risk is comparable to data published by the CDC and explained by the fact that older children and adolescents socialize more than toddler and younger children (43). Interestingly, the highest CFR (Table 2) was observed among children younger than one year of age, having a higher likelihood of dying than older children (male OR: 12. 5; 7.88–19.75, and female OR: 14.3; 8.63–23.66). These findings are similar to other reports (30, 44). Harwood et al. (2022), taking as a reference children from one to four years of age, found that infants (one year and under) are at increased risk of death (OR: 2.08; 1.57–2.86) (42).

The influence of ethnicity on children infected with COVID-19 has not been extensively studied. Oliveira et al. reported that when taking white children as a reference, children of indigenous ethnicity had a higher risk of dying from COVID-19 (HR: 3.36; 2.15–5.24) (28). In this context, we decided to take children of mestizo ethnicity as a reference because it is the most representative ethnic group in Ecuador (45). We found that male indigenous children are at higher risk of dying when compared to other groups (OR: 3.3; 1.71–6.38). This may be due to different factors, including socioeconomic and educational dynamics among this group. In 2018, Ecuador reported that 50.6% of its indigenous population lived in poverty, compared to 20.9% of the non-indigenous population (24). Nationwide data have also reported a 30% higher mortality and 63% higher hospital admission rates than their non-indigenous counterparts (46). In a recently published work, Ortiz-Prado et al., 2022 have found a 12 times greater risk of dying due to water borne diseases among indigenous children when compared them to self-determined white populations (47). Another important factor is the cultural barrier that indigenous people face. Indigenous groups have ancestral practices and views away from doctors, generating conflict between the traditional healer's believe who has the respect of the community and the tradition doctor who may sound distant, arrogant, and detached from the tribe's worldview (48). This could be partly associated with the massive contagion in Ecuador that also affected indigenous groups, regardless of their province or canton of residence (48).

Two of the three provinces with the highest number of COVID-19 cases belong to the coastal region of Ecuador (Guayas and Manabí), we believe that this observation is probably due to the massive contagion that occurred in the coastal region in the initial stage of the pandemic (25). Within the same context, as we have mentioned, we also consider that the highest rates of COVID-19 infection noted in the specific region of Ecuador was due to the largest number of tests that have been performed in provinces such as of Pichincha, Guayas, and Manabí, the ones close to major urban areas in Ecuador such as Quito, Guayaquil, and Manta, respectively. Additionally, to this, the highest number of deaths per 100,000 inhabitants are in urban-marginal or rural cantons, where the most severe cases probably ended in higher hospitalization and mortality rates as well as superspreading events (48–50).

The low prevalence of COVID-19 infection among children in Ecuador is consistent with the findings of previous studies from other countries, suggesting that children are less susceptible to COVID-19 infection than adults (51). However, the study also found that children with comorbidities were more likely to develop severe disease, indicating that children with underlying health conditions should be closely monitored for COVID-19 infection.

To reduce the impact of COVID-19 on vulnerable populations in Ecuador, several recommendations can be implemented. First, healthcare access in rural areas should be improved, and healthcare facilities should be equipped with necessary resources. Second, targeted public health interventions, such as outreach programs, health education, and culturally sensitive messaging, should be implemented to reduce disparities in infection and mortality rates among children with comorbidities and those belonging to indigenous ethnic groups. Third, a robust surveillance and reporting system should be implemented to track the spread of the virus accurately. Fourth, vaccination campaigns should prioritize the most vulnerable populations, such as children under one year of age, those with comorbidities, and those belonging to indigenous ethnic groups. Finally, policies tackling poverty, social exclusion, and access to basic services such as water and sanitation should be implemented to address systemic inequalities that put vulnerable populations at a higher risk of infection and mortality.

## 5. Limitations

While our study provides important insights into the clinical characteristics of pediatric COVID-19 cases in Ecuador, it is important to acknowledge several inherent limitations in its design. One such limitation is the potential underestimation of asymptomatic cases, as our analysis relied solely on reported symptoms. However, it should be noted that during the early months of the pandemic in Ecuador, diagnostic testing was required as part of the healthcare process, which may have reduced the likelihood of missing asymptomatic cases. Another limitation is the lack of information on comorbidities and length of hospital stay for the included children, which may have influenced our findings. Despite these limitations, our study provides valuable information on the presentation and outcomes of pediatric COVID-19 cases in Ecuador, highlighting the need for further research in this area.

Limitations such as underreporting of COVID-19 cases, inadequate diagnostic capability, and disease misclassification could impact the accuracy and validity of our conclusions. We recognize the need to acknowledge and address these limitations to ensure the reliability of our study findings. In future research, we will strive to ensure comprehensive reporting of COVID-19 cases, accurate diagnosis, and proper disease classification to reduce the risk of bias.

Another limitation is the possibility of false positive and false negative results, which could have led to misclassification of patients as either infected or uninfected. Additionally, this study design did not allow for a follow-up process of the registered cases, which would have been optimal to understand the long-term outcomes and potential sequelae of COVID-19 in the pediatric population. Therefore, our findings should be considered as descriptive only, and future studies should aim to address these limitations by using more accurate diagnostic tests and incorporating follow-up assessments to better understand the impact of COVID-19 on pediatric patients.

## 6. Conclusion

This study, the first of its kind in Ecuador, reveals that children have a high likelihood of contracting SARS-CoV-2, with those under one year of age facing the highest risk of mortality. Furthermore, our findings highlight significant disparities in infection and mortality rates among children living in rural areas, those with comorbidities, and those belonging to indigenous ethnic groups. The observed variations in mortality and infection rates across cantons and provinces of Ecuador suggest the existence of systemic inequalities, particularly in impoverished rural regions of the Coast and Amazon areas. These results underscore the need for targeted public health interventions to mitigate the disproportionate impact of COVID-19 on vulnerable populations in Ecuador.

## Data Availability

The original contributions presented in the study are included in the article/Supplementary Material, further inquiries can be directed to the corresponding authors.

## References

[1] Li Q Guan X Wu P Wang X Zhou L Tong Y Early transmission dynamics in Wuhan, China, of novel coronavirus–infected pneumonia. N Engl J Med. (2020) 382:1199–207. 10.1056/NEJMoa2001316 31995857 PMC7121484

[2] CDC. Excess Deaths Associated with COVID-19 (2022). Available at: https://www.cdc.gov/nchs/nvss/vsrr/covid19/excess_deaths.htm (Accessed December 28, 2022).

[3] Johns Hopkins Coronavirus Resource Center. COVID-19 Map. Johns Hopkins Coronavirus Resource Center. Available at: https://coronavirus.jhu.edu/map.html (Accessed December 28, 2022).

[4] Ortiz-Prado E Fernandez R Vascones JE Simbana-Rivera K Correa-Sancho T Lister A Analysis of excess mortality data at different altitudes during the COVID-19 outbreak in Ecuador. High Alt Med Biol. (2021) 22:406–16. 10.1089/ham.2021.0070 34905395

[5] Ortiz-Prado E Fernández-Naranjo R. One Health Research Group, Facultad de Medicina, Universidad de las Américas, Quito, Ecuador. Impacto de la COVID-19 en el Ecuador: De los datos inexactos a las muertes en exceso. Rev Ecuat Neurol. (2020) 29:8–11. 10.46997/revecuatneurol29200008

[6] Thakur B Dubey P Benitez J Torres JP Reddy S Shokar N A systematic review and meta-analysis of geographic differences in comorbidities and associated severity and mortality among individuals with COVID-19. Sci Rep. (2021) 11:8562. 10.1038/s41598-021-88130-w 33879826 PMC8058064

[7] Johnson AG Amin AB Ali AR Hoots B Cadwell BL Arora S COVID-19 Incidence and death rates among unvaccinated and fully vaccinated adults with and without booster doses during periods of Delta and omicron variant emergence — 25 U.S. Jurisdictions, April 4–December 25, 2021. Morb Mortal Wkly Rep (2022):71:132–8. 10.15585/mmwr.mm7104e2 PMC9351531 35085223

[8] Irwin M Lazarevic B Soled D Adesman A. The COVID-19 pandemic and its potential enduring impact on children. Curr Opin Pediatr. (2022) 34:107–15. 10.1097/MOP.0000000000001097 34923563 PMC8728751

[9] Freitas AF Pugliese RPS Feier F Miura IK Danesi VLB Oliveira EN Impact of COVID-19 infection on children and adolescents after liver transplantation in a Latin American reference center. Microorganisms. (2022) 10:1030. 10.3390/microorganisms10051030 35630472 PMC9143523

[10] Borrelli M Corcione A Castellano F Fiori Nastro F Santamaria F. Coronavirus disease 2019 in children. Front Pediatr. (2021) 9:668484. 10.3389/fped.2021.668484 34123972 PMC8193095

[11] Chiwandire N Jassat W Groome M Kufa T Walaza S Wolter N Changing epidemiology of COVID-19 in children and adolescents over four successive epidemic waves in South Africa. J Pediatric Infect Dis Soc. (2023) piad002. 10.1093/jpids/piad002 36648247 PMC10112681

[12] Dong Y Mo X Hu Y Qi X Jiang F Jiang Z Epidemiology of COVID-19 among children in China. Pediatrics. (2020) 145:e20200702. 10.1542/peds.2020-0702 32179660

[13] Liguoro I Pilotto C Bonanni M Ferrari ME Pusiol A Nocerino A SARS-COV-2 infection in children and newborns: a systematic review. Eur J Pediatr. (2020) 179:1029–46. 10.1007/s00431-020-03684-7 32424745 PMC7234446

[14] Ludvigsson JF. Systematic review of COVID-19 in children shows milder cases and a better prognosis than adults. Acta Paediatr Oslo Nor 1992. (2020) 109:1088–95. 10.1111/apa.15270 PMC7228328 32202343

[15] Rudan I Adeloye D Katikireddi SV Murray J Simpson C Shah SA The COVID-19 pandemic in children and young people during 2020-2021: learning about clinical presentation, patterns of spread, viral load, diagnosis and treatment. J Glob Health. (2021) 11:01010. 10.7189/jogh.11.01010 35047182 PMC8763336

[16] UNICEF. Child mortality and COVID-19. UNICEF DATA (2023). Available at: https://data.unicef.org/topic/child-survival/covid-19/ (Accessed March 4, 2023).

[17] CDC. Provisional COVID-19 Deaths: Focus on Ages 0-18 (2022). Available at: https://data.cdc.gov/NCHS/Provisional-COVID-19-Deaths-Focus-on-Ages-0-18-Yea/nr4s-juj3 (Accessed December 28, 2022).

[18] Rabinowicz S Leshem E Pessach IM. COVID-19 in the pediatric population-review and current evidence. Curr Infect Dis Rep. (2020) 22:29. 10.1007/s11908-020-00739-6 32982599 PMC7501762

[19] Patel NA. Pediatric COVID-19: systematic review of the literature. Am J Otolaryngol. (2020) 41:102573. 10.1016/j.amjoto.2020.102573 32531620 PMC7833675

[20] Viner RM Ward JL Hudson LD Ashe M Patel SV Hargreaves D Systematic review of reviews of symptoms and signs of COVID-19 in children and adolescents. Arch Dis Child. (2020). archdischild-2020-320972. 10.1136/archdischild-2020-320972 33334728

[21] Rathore V Galhotra A Pal R Sahu KK. COVID-19 Pandemic and children: a review. J Pediatr Pharmacol Ther. (2020) 25:574–85. 10.5863/1551-6776-25.7.574 33041712 PMC7541032

[22] Kitano T Kitano M Krueger C Jamal H Al Rawahi H Lee-Krueger R The differential impact of pediatric COVID-19 between high-income countries and low- and middle-income countries: a systematic review of fatality and ICU admission in children worldwide. PLoS One. (2021) 16:e0246326. 10.1371/journal.pone.0246326 33513204 PMC7845974

[23] Shekerdemian LS Mahmood NR Wolfe KK Riggs BJ Ross CE McKiernan CA Characteristics and outcomes of children with coronavirus disease 2019 (COVID-19) infection admitted to US and Canadian pediatric intensive care units. JAMA Pediatr. (2020) 174:1–6. 10.1001/jamapediatrics.2020.1948 32392288 PMC7489842

[24] Instituto Nacional de Estadística yCensos. Nacidos Vivos y Defunciones Fetales. Instituto Nacional de Estadística y Censos. Available at: https://www.ecuadorencifras.gob.ec/nacidos-vivos-y-defunciones-fetales/ (Accessed December 28, 2022).

[25] Ortiz-Prado E Simbaña-Rivera K Barreno LG Diaz AM Barreto A Moyano C Epidemiological, socio-demographic and clinical features of the early phase of the COVID-19 epidemic in Ecuador. PLoS Negl Trop Dis. (2021) 15:e0008958. 10.1371/journal.pntd.0008958 33395425 PMC7817051

[26] Moreira A Chorath K Rajasekaran K Burmeister F Ahmed M Moreira A. Demographic predictors of hospitalization and mortality in US children with COVID-19. Eur J Pediatr. (2021) 180:1659–63. 10.1007/s00431-021-03955-x 33474580 PMC7817069

[27] Preston LE Chevinsky JR Kompaniyets L Lavery AM Kimball A Boehmer TK Characteristics and disease severity of US children and adolescents diagnosed with COVID-19. JAMA Netw Open. (2021) 4:e215298. 10.1001/jamanetworkopen.2021.5298 33835179 PMC8035649

[28] Oliveira EA Colosimo EA Silva ACS e Mak RH Martelli DB Silva LR Clinical characteristics and risk factors for death among hospitalised children and adolescents with COVID-19 in Brazil: an analysis of a nationwide database. Lancet Child Adolesc Health. (2021) 5:559–68. 10.1016/S2352-4642(21)00134-6 34119027 PMC8192298

[29] Bolaños-Almeida CE Espitia Segura OM. Clinical and epidemiologic analysis of COVID-19 children cases in Colombia PEDIACOVID. Pediatr Infect Dis J. (2021) 40:e7. 10.1097/INF.0000000000002952 33093428

[30] García-Vera C Castejón-Ramírez S Laín Miranda E Hernández Abadía R García Ventura M Borque Navarro E COVID-19 in children: clinical and epidemiological spectrum in the community. Eur J Pediatr. (2022) 181:1235–42. 10.1007/s00431-021-04235-4 34406504 PMC8371417

[31] Bellino S Punzo O Rota MC Del Manso M Urdiales AM Andrianou X COVID-19 Disease severity risk factors for pediatric patients in Italy. Pediatrics. (2020) 146:e2020009399. 10.1542/peds.2020-009399 32665373

[32] Re3data.Org. The GISAID Initiative (2022). 10.17616/R3Q59F

[33] Ortiz-Prado E Simbaña-Rivera K Gómez-Barreno L Rubio-Neira M Guaman LP Kyriakidis NC Clinical, molecular and epidemiological characterization of the SARS-CoV2 virus and the coronavirus disease 2019 (COVID-19), a comprehensive literature review. Diagn Microbiol Infect Dis. (2020) 98(1):115094. 10.1016/j.diagmicrobio.2020.115094 32623267 PMC7260568

[34] Sippy R Prado EO Pizarro Fajardo F Hidalgo I Aguilar GV Bonville CA Medically attended outpatient coronavirus infections in ecuadorean children during the 20 months preceding countrywide lockdown related to the SARS-CoV-2 pandemic of 2020. Pediatr Infect Dis J. (2020) 39:e291–6. 10.1097/INF.0000000000002840 32773657

[35] Suryadevara M Fajardo FP Aponte CC Carrillo Aponte JL Prado EO Hidalgo I Etiologies of outpatient medically attended acute respiratory infections among young Ecuadorian children prior to the start of the 2020 SARS-CoV-2 pandemic. Influenza Other Respir Viruses. (2023) 17:e13056. 10.1111/irv.13056 36172889 PMC9537809

[36] Zimmermann P Curtis N. Why is COVID-19 less severe in children? A review of the proposed mechanisms underlying the age-related difference in severity of SARS-CoV-2 infections. Arch Dis Child (2021):106:429–39. 10.1136/archdischild-2020-320338 33262177

[37] Castagnoli R Votto M Licari A Brambilla I Bruno R Perlini S Severe acute respiratory syndrome coronavirus 2 (SARS-CoV-2) infection in children and adolescents: a systematic review. JAMA Pediatr. (2020) 174:882–9. 10.1001/jamapediatrics.2020.1467 32320004

[38] Alkhouli M Nanjundappa A Annie F Bates MC Bhatt DL. Sex differences in case fatality rate of COVID-19: insights from a multinational registry. Mayo Clin Proc. (2020) 95:1613–20. 10.1016/j.mayocp.2020.05.014 32753136 PMC7256502

[39] Fortunato F Martinelli D Caputo SL Santantonio T Dattoli V Lopalco PL Sex and gender differences in COVID-19: an Italian local register-based study. BMJ Open. (2021) 11:e051506. 10.1136/bmjopen-2021-051506 34620662 PMC8507404

[40] Galíndez ME Drummond T Rodríguez B Rojas MF Galvis Y Stanchieri M Caracterización clínico epidemiológica de niños con sospecha de la COVID-19 en el hospital universitario de Caracas. Bol Venez Infectol. (2020) 31:9. https://medisur.sld.cu/index.php/medisur/article/view/5418.

[41] Panahi L Amiri M Pouy S. Clinical characteristics of COVID-19 infection in newborns and pediatrics: a systematic review. Arch Acad Emerg Med. (2020) 8:e50. Available from: https://doi.org/https://www.ncbi.nlm.nih.gov/pmc/articles/PMC7212072/32440661 PMC7212072

[42] Harwood R Yan H Camara NTD Smith C Ward J Tudur-Smith C Which children and young people are at higher risk of severe disease and death after hospitalisation with SARS-CoV-2 infection in children and young people: a systematic review and individual patient meta-analysis. eClinicalMedicine. (2022) 44:101287. 10.1016/j.eclinm.2022.101287 35169689 PMC8832134

[43] CDC. Provisional COVID-19 Deaths: Focus on Ages 0-18 Years | Data | Centers for Disease Control and Prevention (2022). Available at: https://data.cdc.gov/NCHS/Provisional-COVID-19-Deaths-Focus-on-Ages-0-18-Yea/nr4s-juj3 (Accessed September 20, 2022).

[44] Sena GR Lima TPF Vidal SA Duarte M do CMB Bezerra PGM Lima EJF Clinical characteristics and mortality profile of COVID-19 patients aged less than 20 years old in pernambuco—brazil. Am J Trop Med Hyg. (2021) 104:1507–12. 10.4269/ajtmh.20-1368 33606669 PMC8045659

[45] Menéndez J Fuentes O Sempértegui B Grijalva J Martínez AG. Censo 2010 poblacion y vivienda. Available at: https://www.ecuadorencifras.gob.ec/wp-content/descargas/Libros/Memorias/memorias_censo_2010.pdf

[46] INEC. Instituto Nacional de Estadisticas y Censos. Available at: https://www.ecuadorencifras.gob.ec/institucional/home/ (Accessed December 28, 2022).

[47] Ortiz-Prado E Simbaña-Rivera K Cevallos G Gómez-Barreno L Cevallos D Lister A Waterborne diseases and ethnic-related disparities: a 10 years nationwide mortality and burden of disease analysis from Ecuador. Front Public Health. (2022) 10:1029375. 10.3389/fpubh.2022.1029375 (Accessed December 28, 2022). 36620267 PMC9811003

[48] Henriquez-Trujillo AR Ortiz-Prado E Rivera-Olivero IA Nenquimo N Tapia A Anderson M COVID-19 outbreaks among isolated amazonian indigenous people, Ecuador. Bull World Health Organ. (2021) 99:478. 10.2471/BLT.20.283028 34248216 PMC8243033

[49] Ortiz-Prado E Henriquez-Trujillo AR Rivera-Olivero IA Freire-Paspuel B Vallejo-Janeta AP Lozada T Massive SARS-CoV-2 RT-PCR testing on rural communities in manabi province (Ecuador) reveals severe COVID-19 outbreaks. Am J Trop Med Hyg. (2021) 104:1493–4. 10.4269/ajtmh.20-1208 33556041 PMC8045655

[50] Rodriguez-Paredes MB Vallejo-Janeta PA Morales-Jadan D Freire-Paspuel B Ortiz-Prado E Henriquez-Trujillo AR COVID-19 Community transmission and super spreaders in rural villages from manabi province in the coastal region of Ecuador assessed by massive testing of community-dwelling population. Am J Trop Med Hyg. (2022) 106:121–6. 10.4269/ajtmh.21-0582 PMC8733526 34788738

[51] Lee PI Hu YL Chen PY Huang YC Hsueh PR. Are children less susceptible to COVID-19? J Microbiol Immunol Infect. (2020) 53:371. 10.1016/j.jmii.2020.02.011 32147409 PMC7102573

