# Supplementary material for: The deadly impact of COVID-19 among children from Latin America: The case of Ecuador
Source: Front Pediatr. 2023 Apr 21;11:1060311. doi: 10.3389/fped.2023.1060311 (PMC10160383; doi:10.3389/fped.2023.1060311)
Supplement: Supplementary file 1 [file Table1.docx]

Supplementary file 1. Analysis of risk and probability of infection and death among Ecuadorian population with COVID-19

|  |  | Deaths | Cases | CFR% | OR | CI <95% | CI>95% | P Value |
| --- | --- | --- | --- | --- | --- | --- | --- | --- |
| 0-4 Years | Female | 70 | 2043 | 3.4% | 12.48 | 7.88 | 19.75 | 0.0001 |
|  | Male | 77 | 2285 | 3.4% | 14.2925 | 8.63 | 23.66 | 0.0001 |
|  |  |  |  |  |  |  |  |  |
| 5 to 9 Years | Female | 17 | 2214 | 0.8% | 2.72 | 1.46 | 5.05 | 0.0015 |
|  | Male | 16 | 2375 | 0.7% | 2.77 | 1.42 | 5.41 | 0.0026 |
|  |  |  |  |  |  |  |  |  |
| 10 to 14 Years | Female | 15 | 4258 | 0.4% | 1.24 | 0.65 | 2.36 | 0.5 |
|  | Male | 19 | 4199 | 0.5% | 1.86 | 0.98 | 3.52 | 0.055 |
|  |  |  |  |  |  |  |  |  |
| 15 to 19 Years | Female | 25 | 8821 | 0.3% | ref | ref | ref | ref |
|  | Male | 19 | 7806 | 0.2% | ref | ref | ref | ref |
|  |  |  |  |  |  |  |  |  |
| 20 to 24 years | Female | 31 | 16700 | 0.2% | 0.65 | 0.38 | 1.1 | 0.115 |
|  | Male | 48 | 15848 | 0.3% | 1.24 | 0.73 | 2.11 | 0.41 |
|  |  |  |  |  |  |  |  |  |
| 25 to 29 Years | Female | 54 | 27540 | 0.2% | 0.69 | 0.43 | 1.111 | 0.127 |
|  | Male | 82 | 26459 | 0.3% | 1.27 | 0.77 | 2.1 | 0.34 |
|  |  |  |  |  |  |  |  |  |
| 30 to 34 years | Female | 93 | 29058 | 0.3% | 1.12 | 0.72 | 1.75 | 0.588 |
|  | Male | 160 | 30204 | 0.5% | 2.18 | 1.35 | 3.5 | 0.0013 |
|  |  |  |  |  |  |  |  |  |
| 35 to 39 Years | Female | 128 | 25378 | 0.5% | 1.78 | 1.16 | 2.73 | 0.0082 |
|  | Male | 238 | 28222 | 0.8% | 3.48 | 2.18 | 5.56 | 0.0001 |
|  |  |  |  |  |  |  |  |  |
| 40 to 44 Years | Female | 188 | 21578 | 0.9% | 3.09 | 2.03 | 4.69 | 0.0001 |
|  | Male | 406 | 24166 | 1.7% | 7.6 | 4.41 | 11.1 | 0.0001 |
|  |  |  |  |  |  |  |  |  |
| 45 to 49 Years | Female | 291 | 18806 | 1.5% | 5.52 | 3.6 | 8.32 | 0.0001 |
|  | Male | 653 | 20282 | 3.2% | 13.63 | 8.63 | 21.5 | 0.0001 |
|  |  |  |  |  |  |  |  |  |
| 50 to 54 Years | Female | 411 | 17272 | 2.4% | 8.57 | 5.7 | 12.8 | 0.0001 |
|  | Male | 1004 | 18337 | 5.5% | 23.7 | 15.06 | 37.4 | 0.0001 |
|  |  |  |  |  |  |  |  |  |
| 55 to 59 Years | Female | 697 | 15883 | 4.4% | 16.14 | 10.8 | 24.08 | 0.0001 |
|  | Male | 1431 | 16895 | 8.5% | 37.92 | 24.09 | 59.6 | 0.0001 |
|  |  |  |  |  |  |  |  |  |
| 60 to 64 Years | Female | 1009 | 12216 | 8.3% | 31.67 | 21.2 | 47.15 | 0.0001 |
|  | Male | 1923 | 13744 | 14.0% | 66.67 | 42.3 | 104.8 | 0.0001 |
|  |  |  |  |  |  |  |  |  |
|  |  |  |  |  |  |  |  |  |
| 65 to 69 Years | Female | 1222 | 9294 | 13.1% | 53.26 | 35.8 | 79.2 | 0.0001 |
|  | Male | 2166 | 10954 | 19.8% | 101.01 | 64.2 | 158.8 | 0.0001 |
|  |  |  |  |  |  |  |  |  |
| 70 to 74 Years | Female | 1180 | 6699 | 17.6% | 75.22 | 50.54 | 111.95 | 0.0001 |
|  | Male | 2181 | 8384 | 26.0% | 144.1 | 91.6 | 226.6 | 0.0001 |
|  |  |  |  |  |  |  |  |  |
| 75 to 79 Years | Female | 1022 | 4776 | 21.4% | 95.78 | 64.29 | 142.69 | 0.0001 |
|  | Male | 1849 | 6061 | 30.5% | 179.9 | 114.3 | 283.1 | 0.00001 |
|  |  |  |  |  |  |  |  |  |
| > 80 Years | Female | 1994 | 7139 | 27.9% | 136.35 | 91.7 | 202.6 | 0.0001 |
|  | Male | 3084 | 8041 | 38.4% | 254.9 | 162.1 | 400.87 | 0.00001 |
