# Supplementary figures and images for: The deadly impact of COVID-19 among children from Latin America: The case of Ecuador
Source: Front Pediatr. 2023 Apr 21;11:1060311. doi: 10.3389/fped.2023.1060311 (PMC10160383; doi:10.3389/fped.2023.1060311)

Supplementary file 2. Circulating SARS-CoV-2 virus variants in Ecuador from April 2020 to May 2022


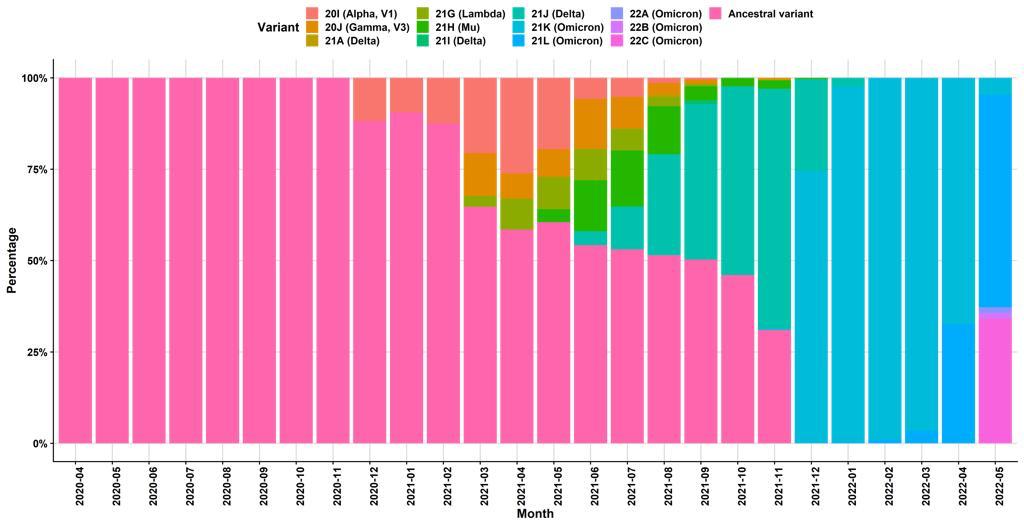

Supplement: Supplementary file 2 [file Table2.docx]
